# The Doctors' Decision

**Published:** 1912-12-28

**Authors:** 


### THE DOCTORS' DECISION.

It has been obvious for a long time that the general current of feeling in the medical profession is strongly opposed to acceptance of Mr. George's revised terms for medical attendance under the Insurance Act. For our own part, as outsiders, we expressed the opinion that the terms were almost good enough, and that if certain modifications about extras, mileage, and control were conceded, the doctors would be well advised to accept them. To that opinion we still adhere; but since none of the modifications have been granted, and the Chancellor seems to have hardened his heart entirely, we quite agree with the British Medical Association in their refusal to countenance service under the Act.

The situation at the present moment is serious enough, in all conscience, to engage the close attention of every citizen this Christmastide. Some two thousand four hundred members of the medical profession voted in favour of accepting the proffered terms, and about half the total number of the doctors did not vote at all. What prospect is there of the panels being adequately staffed? To begin with, it is not safe to conclude that the two thousand four hundred will go "on the panel." Suppose a thousand of them do follow the lead of Mr. Lloyd George's friends, Doctors Addison, Shaw, Mills, and Co. Does it follow that the unpolled moiety of the profession will furnish a similar number? We doubt it; but assume that such will be the case. Two thousand doctors—the majority of them, it is safe to say, of the lower levels of professional efficiency—for fourteen million insured. In some few districts it may be possible to staff the panel properly, but in the great majority it certainly will not be. If on January 15 there is no medical benefit for the insured, it is the Chancellor's doing, and no one else's.

The Labour Party may be trusted to appreciate this point, since their constituents will doubtless press it upon their attention. With the exception of "my friend Lansbury," the Labour Party swallowed the Act without taking the trouble to examine it carefully or to think out its eventual effects. Without their co-operation it could not have been rushed as it was through Parliament with three-quarters of its clauses undiscussed. The Labour members cannot, therefore, shirk responsibility for its effects. One of those effects will be—nay, already is—the weakening of trades unions. It is true that these organisations are qualified to be "approved societies" under the Act, provided they conduct the insurance work as an entirely separate department. But it is already obvious that the great friendly societies and such assurance companies as the Prudential can offer far better benefits than the trades unions can, and they will inevitably attract the bulk of the insured in consequence.

Another interesting problem for Labour members is their attitude towards the doctors. The Chancellor is doing his level best to staff his panels with what the Labour-Socialist party would call "blackleg labour." He is, further, endeavouring to attract "strike-breakers" by promises that they will receive preference in respect of whole-time appointments under the Act. We all know what the Labour Party would do were a member of the Government to connive at, much less to adopt, such methods of dealing with an industrial dispute. One of their spokesmen is even now advocating a general strike to secure an eight-hours' day. What about the doctors? Does anyone imagine their "panel" work will be done in eight hours?

The doctors, however, are not content merely to refuse to co-operate with the Chancellor on his own terms. They have, through their organisation, the British Medical Association, already prepared a scheme of their own for attending the sick poor on their own terms. It would appear that those terms, financially, are no more expensive to the insurance societies than Mr. George's; this disposes effectually of the taunt that the Association is out for mere lucre. In other respects the difference is great; for instance, over the matter of lay control, which has stuck in the doctors' gizzards more firmly than any other of the Chancellor's refreshing fruits.

